# Modeling the Intervention of HIV Transmission across Intertwined Key Populations

**DOI:** 10.1038/s41598-018-20864-6

**Published:** 2018-02-05

**Authors:** Lu Zhong, Qingpeng Zhang, Xiaoming Li

**Affiliations:** 1 Department of Systems Engineering and Engineering Management, City University of Hong Kong, Kowloon, Hong Kong, SAR China; 2 Shenzhen Research Institute of City University of Hong Kong, Shenzhen, Guangdong, China; 3 Arnold School of Public Health, University of South Carolina, Columbia, SC USA

## Abstract

The HIV transmissions between multiple key populations make interventions difficult, particularly with multiple transmission behaviors. It remains unclear how significant the role of bridge individuals (who connect multiple communities) is in HIV transmission, and how to develop more effective intervention strategies targeting different transmission modes across key populations. In this research, we proposed a 2-layer social network framework to simulate the HIV transmissions across female sex workers (FSWs) and persons who inject drugs (PWID) through two behaviors: unprotected sex and needle-sharing. We proposed a set of intervention strategies based on the topological properties of individuals in the social network and estimated the efficacy of these strategies. Simulation studies demonstrated that bridge individuals played a significant role in HIV transmissions across the two networks. Prevention on such bridge individuals could help reduce both the scale and speed of HIV transmissions.

## Introduction

HIV (Human immunodeficiency virus) is a critical health threat and healthcare burden worldwide^1^. HIV spreads through transferring bodily fluid in multiple ways, including unprotected sex, transfusing blood, sharing needles, mother-to-infant transmission, and breastfeeding^2^. Common HIV prevention intervention strategies include behavioral interventions, biomedical interventions, and structural interventions^3–5^. Behavioral research has unveiled that the interactions between female sex workers (FSWs) and persons who inject drugs (PWID) could facilitate the transmission of HIV among these two key populations^6–9^. Recent field studies identified the high prevalence of intimate relationships between FSWs and PWID^10–14^. For example, many FSWs are found to have noncommercial PWID partners, because of the match between of FSWs’ needs for emotional support and PWID’s needs for financial support^15–17^. Such overlapping of these two key populations boosts the spread of HIV infections, resulting in a huge impact on HIV preventions^18,19^. Controlling “bridges” connecting two populations is a possible way to improve the efficiency and efficacy of prevention program, particularly in low and middle-income countries (LMIC)^9,20,21^, where limited resource are available to urgently needed HIV prevention programs^22^.

Social networks play an important role in characterizing and modeling the epidemics of infectious diseases like HIV^23–26^. Most social network studies of HIV relied on small-scale survey and investigations, which could lead to biased data samples and unreliable results. In addition, existing studies mainly adopted single-layer network to represent the relations and disease transmissions between people^27^. This approach simplifies the complex heterogeneous relations with only one type of edge in the network^28^. However, in the real-world case, there are usually multiple types of relations through which infectious diseases transmit^29^. For example, field studies revealed that HIV is mainly transmitted through having unprotected sex, and sharing injection equipment while taking drugs^30^. These two types of relationships exhibit different transmission patterns of HIV, making the classic single-layer network model incapable of representing the epidemics patterns accurately. To the best of our knowledge, there is no modeling research quantifying the efficacy of intervention strategies in such intertwined key populations with multiple relationships in the social network.

To address this challenge, we investigated the importance of bridge individuals who connected two key populations via two modes of HIV transmissions and develop a simulation model to evaluate the efficacy of multiple intervention strategies in large-scale social networks. To better control the HIV transmission across two communities, we proposed a novel cross-layer betweenness centrality metric in the 2-layer network to measure the importance of bridge individuals. We performed simulations to demonstrate the efficacy of intervention proposed strategies based on the topological properties of the 2-layer network.

## Results

In this section, we first describe the proposed 2-layer network framework and intervention strategies based on its topological properties, and present the simulation results. Details of network constructions and calculations of topological properties are introduced in the Methods section.

### 2-layer network framework

We constructed a 2-layer social network to represent the HIV transmissions among two key populations – FSWs and PWID. The two layers of the network represent the HIV transmission through unprotected sex and needle sharing, respectively. Based on the proposed 2-layer social network framework, we adapted the Susceptible-Infected model to simulate HIV transmissions, and quantitatively evaluate the efficacy of multiple structural intervention strategies on HIV transmission.

**Figure 1:**
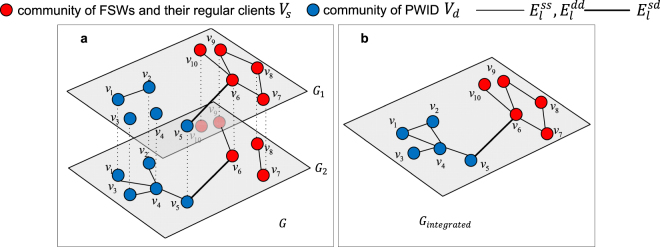
An example of the 2-layer network framework and its integrated network. (**a**) In the 2-layer network framework, layer ${G}_{1}$ and layer ${G}_{2}$ represent the relationships of unprotected sex and needle-sharing between individuals, respectively. Red nodes represent the individuals in the community of FSWs and their regular clients. Blue nodes represent the individuals in the community of PWID. The edges between the two communities are highlighted in bold, representing bridges. (**b**) The integrated network of (**a**) with the same set of nodes and the union of edges in both layers.

We use $G=(V,{E}_{1},{E}_{2})$ to represent an $2$-layer undirected network. The node and edge sets are denoted as $V=({v}_{1},{v}_{2},\ldots ,{v}_{N})$ and ${E}_{l}=\{{e}_{l}^{i,j}|\forall {v}_{i},{v}_{j}\in V\}$, respectively. Here $l=1,\,2$ denotes the layer. All nodes exist in both layers. There is one type of relationships for HIV transmissions at each layer. The first layer, ${G}_{1}=(V,\,{E}_{1}),\,$represents the relationship of unprotected heterogeneous sex. The second layer, ${G}_{2}=(V,{E}_{2}),$ represents the relationship of needle-sharing in taking drugs. In both layers, people formed two clusters – ${V}_{s}$ represents the community of FSWs and their regular clients, and ${E}_{l}^{ss}$ represents the relationships within this community; Similarly, ${V}_{d}$ represents the community of PWID, and ${E}_{l}^{dd}$ represents the relationships within this community; ${E}_{l}^{sd}$ represents the set of edges bridging the two communities at layer $l$ (we denote such edges as *bridges*). The nodes connected by bridges are defined as *bridge nodes*. The set of bridge nodes is represented by ${V}_{bridge}=\{{v}_{i},{v}_{j}|({v}_{i},{v}_{j})\in {E}_{l}^{sd}\}$. Therefore, $V={V}_{s}{\cup }^{}{V}_{d}$ and ${E}_{l}={E}_{l}^{ss}\,{\cup }^{}{E}_{l}^{dd}{\cup }^{}{E}_{l}^{sd}$. In addition, we also define an integrated network ${G}_{integrated}=(V,E)$, where $E={E}_{1}{\cap }^{}{E}_{2}$. ${G}_{integrated}$ is a single-layer network of the same set of nodes and the union of edges in both layers (excluding duplicate edges that exist in both layers). An example of the framework and the integrated network are given in Fig. 1.

### Network construction

Both the sexual relationship network of FSWs with their regular clients and the needle-sharing relationship network of PWID have been identified to be scale-free^27,31^. It is worth noting that both FSWs and their regular clients follow scale-free distribution, with comparable heavy-tail effect^32,33^. Therefore, we adapted the classic preferential attachment framework to construct the sexual relationships ${E}_{1}^{ss}$, and needle-sharing relationships ${E}_{2}^{dd}$, respectively. In addition, existing studies pointed out that there were usually erratic needle-sharing relationships within the community of FSWs and their regular clients^17^. Similarly, erratic sexual relationships were also found within the community of PWID^34^. Thus, we adapted the random graph model to simulate random sexual relationships ${E}_{1}^{dd}$ (in the community of PWID), and needle-sharing relationships ${E}_{2}^{ss}$ (in the community of FSWs and their regular clients).

It is worth noting that the current models did not take homosexual relationship in the community into consideration. All cycles in the ${G}_{1}$ have even number of edges (even cycles). Cycles with an odd number of edges (odd cycles), on the other hand, are not possible in ${G}_{1}$. Therefore, we modified the classic preferential attachment model and the random graph model to generate the network without odd cycles, making it a two-mode network. Details of the classic models and the modified models were given in the Methods section.

Recent field studies identified the intimate partnership (i.e. sex partners, couples, etc.) between $${V}_{s}$$ and $${V}_{d}$$, thus coupling of the two corresponding communities^15–18^. The coupling was set to be one on one – a node could connect to only one node in the other community (representing formed intimate partnership). Because there are risks of both sexual and drug interactions in such partnership, we assumed the two corresponding nodes to be connected at both layers ($${E}_{1}^{sd}={E}_{2}^{sd})$$.

**Figure 2:**
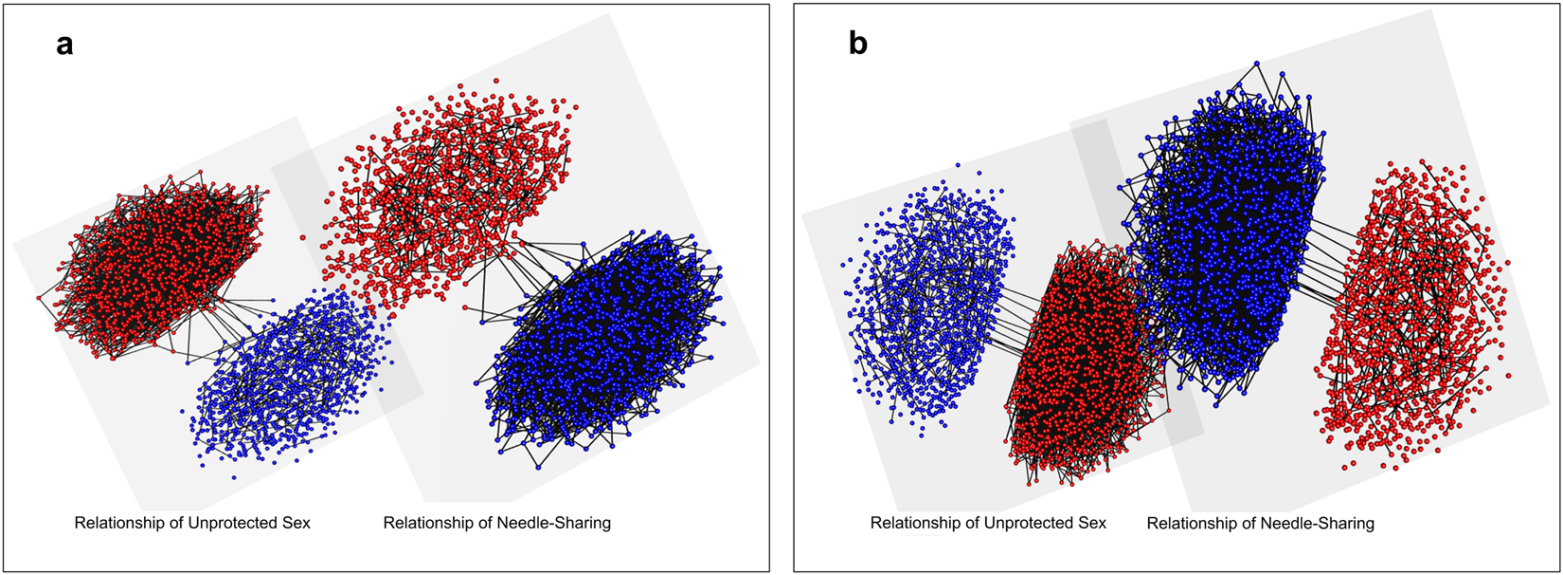
The visualizations of the constructed networks. (**a**) ${G}_{unifrom}$. (**b**) ${G}_{priority}$. Red nodes represent the individuals in the community of FSWs and their regular clients and blue nodes represent the individuals in the community of PWID. Edges between two communities are bridges. Visualizations were generated using *muxViz* software^61^.

Little is known about the characteristics of the bridge nodes ${V}_{bridge}$ that formed relationships with individuals in another community. For generality, we used two approaches to locating the bridge nodes that couple the two communities – *uniform coupling* and *priority coupling*. In the *uniform coupling* approach, individuals ${v}_{i}$ in community ${V}_{s}$ has the uniform probability to be chosen as the bridge node: ${P}_{uniform}({v}_{i})=\frac{1}{{N}_{s}}$, where ${N}_{s}$ is the number of individuals in the community of FSWs and their regular clients. The same criterion is applied to ${v}_{j}$ in community ${V}_{d}$: ${P}_{uniform}({v}_{j})=\frac{1}{{N}_{d}}$, where ${N}_{d}$ is the number of individuals in the community of PWID. In the *priority coupling* approach, the probability of an individual ${v}_{i}$ in community ${V}_{s}$ being chosen as the bridge node is proportional to its degree in the network: ${P}_{priority}({v}_{i})\propto k(i)$, where $k(i)$ represents the degree of ${v}_{i}$ in the integrated network ${G}_{integrated}$. The same criterion is applied to ${v}_{j}$ in community ${V}_{d}$: ${P}_{priority}({v}_{j})\propto k(j)$, where $k(j)$ represents the degree of ${v}_{j}$ in the integrated network ${G}_{integrated}$. With the aforementioned network construction methods, we constructed two networks (Fig. 2). Table 1 summarizes the summary statistics of the networks. We also present the network properties of the bridge individuals in the context of the whole network. In ${G}_{unifrom}$, the bridge individuals were selected randomly, therefore their average degree is close to the average degree of the whole network. In ${G}_{priority}$, the bridge individuals were selected as those with the highest degrees, therefore, their degrees are top 1% in the whole network.

**Table 1:** Networks generated by different coupling approaches.

Network	${\boldsymbol{l}}$	${\boldsymbol{N}}$	${{\boldsymbol{M}}}_{{\boldsymbol{l}}}$	${{\boldsymbol{N}}}_{{\boldsymbol{s}}}$	${{\boldsymbol{M}}}_{{\boldsymbol{l}}}^{{\boldsymbol{ss}}}$	${{\boldsymbol{N}}}_{{\boldsymbol{d}}}$	${{\boldsymbol{M}}}_{{\boldsymbol{l}}}^{{\boldsymbol{dd}}}$	${{\boldsymbol{M}}}_{{\boldsymbol{l}}}^{{\boldsymbol{sd}}}$	$|{{\boldsymbol{V}}}_{{\boldsymbol{bridge}}}|$	<k> in ${{\boldsymbol{V}}}_{{\boldsymbol{bridge}}}$	<k>
${G}_{unifrom}$	$1$	2000	2207	1000	1997	1000	200	10	20	2.25	2.207
	$2$	2000	2207	1000	200	1000	1997	10	20	2.3	2.207
${G}_{priority}$	$1$	2000	2207	1000	1997	1000	200	10	20	21	2.207
	$2$	2000	2207	1000	200	1000	1997	10	20	21.6	2.207

${G}_{unifrom}$ was generated by the uniform coupling approach. ${G}_{priority}$ was generated by the priority coupling approach. $l$ represents the layer and $l=1,2.\,N=|V|$ represents the number of nodes. ${M}_{l}=|{E}_{l}|$ represents the number of edges in ${G}_{l}$. ${N}_{s}=|{V}_{s}|$ and ${M}_{l}^{ss}=|{E}_{l}^{ss}|$ represent the number of nodes and edges in the community of FSWs and their regular clients, respectively. Similarly, ${N}_{d}=|{V}_{d}|$ and ${M}_{l}^{dd}=|{E}_{l}^{dd}|$ represents the number of nodes and edges in the community of PWID, respectively. ${M}_{l}^{sd}=|{E}_{l}^{sd}|$ is the number of bridge edges connecting bridge nodes cross two communities. $|{V}_{bridge}$| is the number of bridge nodes. <k> in ${V}_{bridge}$ represents the averaged degree of bridge nodes. <k> is the averaged degree of nodes in layer $l$.

### Intervention strategies

Targeting individuals holding critical positions in social networks is a common intervention strategy for infectious diseases including HIV^30^. The identification of these target individuals for intervention is primarily based on their rankings in terms of certain topological properties^35^. The most commonly adopted topological ranking criteria are degree centrality and betweenness centrality^36^. Degree centrality quantifies the exposure of an individual in the community by counting the number of neighbors of the corresponding node. Betweenness centrality quantifies the significance of an individual in controlling virus propagation by calculating the proportion of shortest paths traversing through the corresponding nodes^37^. However, these two methods were proposed for the singular network with only one type of edges, while ignoring the existence of multiple relationships between individuals. Therefore, in addition to testing the efficacy of these two conventional singular network based methods, we proposed a novel method named *cross-layer betweenness centrality* to identify target individuals holding a significant role in cross-community HIV transmissions on 2-layer networks.

Details of degree centrality $D({v}_{i})$ and betweenness centrality $B({v}_{i})$ are given in the Methods section. In *degree-based strategy* and *betweenness-based strategy*, we set the priority of nodes being isolated according to their degree and betweenness centralities in ${G}_{integrated}$ – nodes with a higher value of $D({v}_{i})$ or $B({v}_{i})$ will have a higher priority to be isolated in the corresponding strategy.

The conventional *betweenness-based strategy* ignores the significance of cross-layer paths, which are critical for the structure and connectivity of 2-layer networks. In this study, cross-layer paths enable the HIV transmissions by two modes (unprotected sex and needle-sharing), thus indicating the hidden risk across communities that could not be captured by singular network analysis. For example, in Fig. 1, the shortest paths between ${v}_{5}$ and ${v}_{9}$ could traverse edges in one layer: ${e}_{1}^{5,6}$ and ${e}_{1}^{6,9}$ in layer ${G}_{1}$, or ${e}_{2}^{5,6}$ and ${e}_{2}^{6,9}$ in layer ${G}_{2}$. There emerge the cross-layer shortest paths between ${v}_{5}$ and ${v}_{9}$: ${e}_{1}^{5,6}$ and ${e}_{2}^{6,9}$, or ${e}_{2}^{5,6}$ and ${e}_{1}^{6,9}$. Here ${v}_{5}$ and ${v}_{6}$ are bridge nodes because they connect the two communities. The integrated view of the 2-layer network ${G}_{integrated}$ only considers one shortest path between ${v}_{5}$ and ${v}_{9}$ via ${v}_{6}$, while ignores the cross-layer nature of HIV transmissions by multiple modes. Because the two communities have two different main transmission methods, the cross-layer paths indicate the high risk of cross-community transmissions. In order to capture such cross-layer paths and the role of bridge nodes, we propose a new metric, named cross-layer betweenness centrality $Bc({v}_{i})$. Different from $B({v}_{i})$, $Bc({v}_{i})$ does not only measure the influence of a node on HIV transmissions within a layer, but also measures the influence of a node on cross-layer transmissions. The proposed cross-layer betweenness centrality is calculated as follows,

$$Bc({v}_{i})=\sum _{{v}_{i}\ne {v}_{u}\ne {v}_{t}\in V}[\frac{{\delta }_{{v}_{u},{v}_{t}}({v}_{i})}{{\delta }_{{v}_{u},{v}_{t}}}\times (\frac{{\hat{\delta }}_{{v}_{u},{v}_{t}}({v}_{i})}{{\delta }_{{v}_{u},{v}_{t}}}+1)],$$

where ${\delta }_{{v}_{u},{v}_{t}}$ is the total number of the shortest path between ${v}_{u}$ and ${v}_{t}$, ${\delta }_{{v}_{u},{v}_{t}}({v}_{i})$ is the number of the shortest path between ${v}_{u}\,$and ${v}_{t}$ through ${v}_{i}$, ${\hat{\delta }}_{{v}_{u},{v}_{t}}({v}_{i})$ is the number of the cross-layer shortest path between ${v}_{u}\,$and ${v}_{t}$ through ${v}_{i}$. In the proposed *cross-layer betweenness-based strategy*, we set the priority of nodes being isolated according to their cross-layer betweenness value – node with a higher value of $Bc({v}_{i})$ will have a higher priority to be isolated.

As a baseline, we also investigated intervention strategies based on nodes targeted randomly, in which we isolate nodes randomly with the same chance. All the intervention strategies are listed in Table 2.

**Table 2:** Intervention strategies based on multiple metrics.

Strategy	Descriptions
IR	Isolate nodes randomly.
ID	Isolate nodes based on degree centrality (descending order).
IB	Isolate nodes based on betweenness centrality (descending order).
IBc	Isolate nodes based on cross-layer betweenness centrality (descending order).

### The efficacy of intervention strategies

With the constructed 2-layer network framework, we conducted experiments using Susceptible-Infected (SI) simulation model to evaluate the efficacy of proposed intervention strategies. We fixed the value of factors to build a realistic environment for simulation according to the observations from field studies in United States^38^, China^12,15^ and other countries^39^ (details are listed in Methods section). The intervention strategies are evaluated under different values of isolation degree $\alpha \in [0,\,1]$, which is the ratio of isolated people. The efficacy is evaluated by the ratio of infected nodes when the transmission is at the endemic equilibrium. A better intervention strategy is associated with a smaller value of the ratio of infected nodes, indicating a smaller scale of HIV transmission.

**Figure 3:**
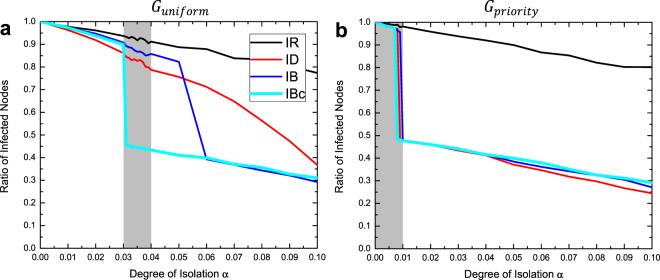
The ratios of infected nodes of intervention strategies (IR, ID, IB, IBc) at the endemic equilibrium with varying degrees of isolation ${\boldsymbol{\alpha }}$ ($0\le {\boldsymbol{\alpha }}\le 0.1$). The dashed area has a higher resolution of $\alpha$ (0.001) because segregations occurred here. (**a**) ${G}_{uniform}$. (**b**) ${G}_{priority}$.

Figure 3 presents the ratio of infected nodes of the four intervention strategies (IR, ID, IB, IBc) at the endemic equilibrium. In general, the ratios of infected nodes for all intervention strategies are declining with an increasing value of $\alpha$ (degree of isolation). This is under expectation because a higher degree of isolation results in a higher chance of breaking the connectivity of the network.

For ${G}_{unifrom}$, whose bridge nodes were selected randomly, we observed a sharp drop (around 50%) of the ratio with increasing $\alpha$ in IB and IBc interventions (Fig. 3a). The sharp drop indicated that we successfully limited the HIV transmission within one community. We name the value of $\alpha$ at such sharp drops as the *segregation point* for each intervention. This segregation of HIV transmission is critical for HIV interventions because we could significantly lower the risk for the vulnerable populations. Simulation results showed that only IB and IBc could achieve such segregation. This is due to the fact that both IB and IBc were based on betweenness centrality, which could help identify bridge nodes. Comparing the two, IBc achieved the segregation point with smaller $\alpha$ than IB (0.031 versus 0.06, 48.3% smaller). When the value of $\alpha$ is larger than the critical segregation point, IB and IBc performed similarly with a much lower ratio of infected nodes.

For ${G}_{priority}$, on the other hand, the chance of a node being selected as a bridge node was proportional to its degree. Therefore, bridge nodes tend to be those with a high degree in the network, making such bridge nodes even more critical for HIV transmission. Therefore, both interventions based on degree centrality (ID) and betweenness centralities (IB and IBc) could achieve the segregation point with a small degree of isolation ($\alpha  < 0.01)$, as shown in Fig. 3b. In particular, IBc with the proposed cross-layer betweenness centrality metric achieved the segregation point earliest out of the three. After the segregation point was captured ($\alpha  > 0.01$), ID, IB, and IBc had comparable performance, while ID had an edge over the other two with a steeper slope.

**Figure 4:**
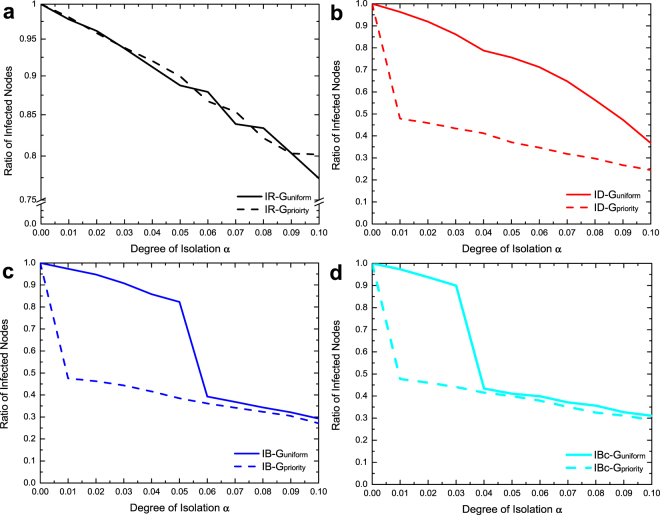
The ratios of infected nodes of intervention strategies (IR, ID, IB, IBc) in ${G}_{uniform}$ and ${G}_{priority}$. (**a**) IR. (**b**) ID. (**c**) IB. (**d**) IBc.

Figure 4 presents the ratios of infected nodes of the four intervention strategies in four corresponding sub-figures, in which the curves of ${G}_{uniform}$ and ${G}_{priority}$ are shown in the same sub-figures. We found that prioritized interventions (ID, IB, and IBc) could achieve the segregation point with a much smaller $\alpha$ in ${G}_{priority}$. In particular, ID could reach the segregation point in ${G}_{priority}$ but not ${G}_{uniform}$ when $\alpha  < 0.1$. The efficacies of IR in both ${G}_{uniform}\,$and ${G}_{priority}$ are similar. These findings indicate that prioritized interventions are more effective if the two communities are coupled by densely connected nodes.

**Figure 5:**
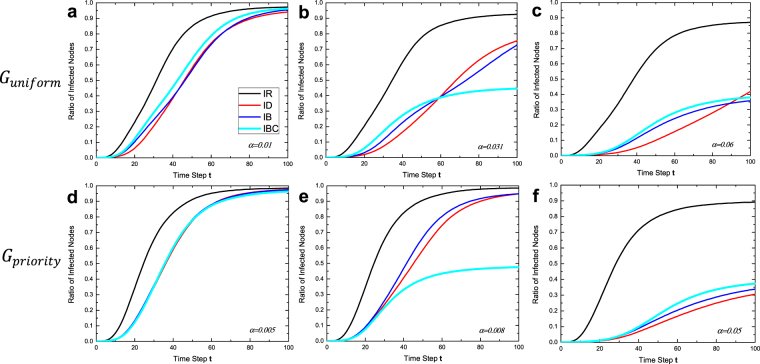
The ratios of infected nodes of interventions strategies (IR, ID, IB, IBc) along with time step ${\boldsymbol{t}}$ at different degrees of isolation ${\boldsymbol{\alpha }}$. (**a**,**d**) No strategies achieve segregation point. (**b**,**e**) Only IBc achieves segregation point. (**c**,**f** ) More strategies achieve segregation points.

The ratios of infected nodes of the four intervention strategies (IR, ID, IB, IBc) over time $t$ are shown in Fig. 5. We selected three values of $\alpha$ for ${G}_{uniform}$ (first row) and ${G}_{priority}$ (second row), respectively, representing three typical outcomes of interventions: no method achieved the segregation point (Fig. 5a,d), IBc achieved the segregation point (Fig. 5b,e), and more than one methods achieved the segregation point (Fig. 5c,f).

When $\alpha$ was small enough, no method could achieve the segregation point, with a fast HIV transmission speed (Fig. 5a,d). As we observed in Fig. 3, IBc was the first method to achieve the segregation point for both ${G}_{uniform}$ and ${G}_{priority}$ when $\alpha$ increases. Under such conditions (Fig. 5b,e), we found that IBc significantly lowered the speed of HIV transmission, which was capped at about 0.5 because the two communities were split apart. When $\alpha$ is large enough that IB and ID also achieved the segregation point, we found the similar results with a slower speed of HIV transmission with IBc, IB, and even ID, without a clear difference in performance (Fig. 5c,f). The slowing transmission speed was mainly caused by the segregation of two communities.

## Discussion

The simulation results consistently showed that it is important to reach the segregation point with a small degree of isolation, so that HIV transmissions could be limited within one community. To do so, we should focus on the *bridge nodes*, which connect two key communities with two modes of HIV transmission. The proposed cross-layer betweenness centrality metric could help achieve the segregation point with a lower degree of isolation, because it tended to prioritize the cross-layer paths traversing through bridge nodes.

This study complements the existing research on modeling HIV transmissions in social networks. The critical role of bridge nodes was validated, indicating the efficacy to prioritize the individuals belonging to multiple key populations in HIV interventions. The findings of this study provided model-based insights for public health researchers and practitioners to develop effective HIV intervention programs, where cross-population interactions through multiple HIV transmission modes exist. This is particularly important for LMIC, where budget and resources for HIV preventions are limited.

For key populations of FSWs and PWID, this research demonstrates the feasibility of reducing the risk of cross-community transmissions via timely interventions in the FSWs and PWID who formed sexual partnerships^40^. It is suggested that public health researchers and social workers identify the high-risk FSWs who are using drugs, and FSWs who have regular sex partners who are using drugs through a venue-based approach^41,42^. Our prior field studies demonstrated that venue-based recruitment and intervention were effective in identifying hard-to-reach populations in China^43^. In practice, social workers could intreview the gatekeeper to identify these high-risk FSWs who or whose partner are using drugs^44^. We can also ask FSWs to identify these high-risk individuals among themselves. After identifying high-risk FSWs, we should (a) inform them the elevated risk of HIV infection caused by unprotected sex and sharing needles, (b) increase their awareness of common modes of HIV transmissions in both communities, (c) assist them to disclose their HIV-related risk to their sexual or drug use partners, (d) help them reduce the risk via providing free condoms and syringes/needles, and the needed information of early Antiretroviral Therapy (ART) initiation, Post-Exposure Prophylaxis (PEP) and Pre-Exposure Prophylaxis (PrEP), and (e) provide HIV testing related information and equipment. In addition to the intervention for high-risk FSWs, it is also suggested that the gatekeepers can play a vital role in educating the FSWs and their clients the risk of HIV transmissions via different modes. For non-bridge FSWs and PWID, this research suggests that individuals in the center of social networks (densely connected) are of higher importance for interventions as compared with those on the margin (less connected). We could identify these densely connected FSWs and PWID using a similar venue-based approach as described above.

Recently, it has been found that by analyzing HIV sequence data, one can identify molecular clusters of HIV cases, and infer the underlying transmission clusters and risk networks, which are very difficult to be fully revealed^45,46^. Existing research with HIV-1 *pol* sequence data demonstrated that the analysis of such data could characterize a highly connected transmission network, based on which network-based risk score can be derived for effective interventions^47^. In this simulation study, we assumed that full information of the risk network was known. In practice, however, it is almost impossible to reveal the complete transmission cluster and the underlying risk network. HIV sequence data and the associated molecular clusters represent a promising way to integrate simulation models with limited real-world data. We can adopt a similar approach using HIV sequence data to identify molecular clusters, transmission clusters, and the underlying risk network for HIV transmissions, so that we can fully utilize the outcomes of this simulation-based research.

This research has several limitations. First, this research did not investigate the effect of network turnovers^48–50^, because we focused on examining the efficacy of structural interventions in a consistent social networking environment. In real-world, social networks are usually dynamic, and the behaviors of individuals could influence others, making the actual interventions much more complicated and unpredictable. In our future research, we will perform field-studies to learn the patterns of network turnovers, and then improve our model with dynamic network features. Second, we did not evaluate the effect of herd immunity^51,52^. Although HIV is not immutable, certain groups of people may become “immutable” because of their behaviors. For example, a non-drug taking FSW could use condoms in all sexual intercourse with regular clients and partners, thus become immutable. Proper use of early ART initiation, PEP, and PrEP could also reduce the risk of infection significantly. A herd immunity may emerge along with the interventions and network turnovers. To keep our model simple and focused on network-based interventions, we did not consider such herd immunity in the population. Third, there are extensive studies on the bridging effect of regular clients of FSWs in transmitting HIV to the general population^21,53–55^. This is an important aspect of HIV transmissions facilitated by FSWs. However, we did not consider this bridging effect in this study since we focused on the interactions between FSWs and PWID. In the future, we plan to develop a three-layer network framework to further integrate the general population into the model. Fourth, large-scale real-world data is almost impossible to get. The simulation models developed in this study were based on descriptive data from frontline interviews and literature, thus with potential noise and bias. As discussed above, we will explore to use HIV sequence data and other mechanisms to reveal the risk network with higher resolution. Last, the efficacy of different intervention strategies could vary at different stages of HIV transmissions and interventions. In the future research, we plan to develop adaptive intervention strategies to take advantages of multiple intervention strategies for better intervention outcome.

## Methods

### Simulation Details

Because (a) there is no cure for HIV, (b) HIV is often with a long incubation period, and (c) infected patients are usually with a relatively long survival time (nine to eleven years), we adopted the Susceptible-Infected (SI) model, in which susceptible nodes (representing healthy individuals) can be infected through interacting with infected nodes (HIV carriers). Once infected, they will not recover nor die (within the simulation time period), and will continuously be capable of spreading the disease to susceptible nodes. In this model, the time unit of simulation is 1 month and the total time the simulation runs is 200-month. The probability of a susceptible node A being infected through interacting with an infected neighbor node B in every month was empirically determined to be ${\beta }_{1}=0.1$ in layer 1 (through the unprotected sexual relationship) and ${\beta }_{2}=0.05$ in layer 2 (through needle-sharing relationship)^39,56^. It is worth noting that in this model, the more infected partners a person has, the more likely he/she will get infected in each month. The risk of infection is constrained by the number of partners the person has (as represented by the degree of the corresponding node in ${G}_{1}$). The risk is not growing linearly with the number partners a person has. It is actually an exponential decay (increasing form) curve, with a limiting value of 1. To account for the randomness of human behaviors, we also did another set of experiments to allow the transmission rate ${\beta }_{1}$ and ${\beta }_{2}$ to vary for different edges following a Gaussian distribution: ${\beta }_{1}\, \sim N(0.1,{\sigma }^{2})$, ${\beta }_{2}\, \sim N(0.05,{\sigma }^{2})$. Simulation results with varying transmission rates are presented in the supplementary materials.

In each simulation run, we randomly chose one individual as the initially infected node, thus the number of infected nodes at time zero ${I}_{0}$ was 1. For each intervention strategy, we set ${I}_{t}$ as the average of the counts of infected nodes at time $t$ in 1000 simulation runs. Without interventions, eventually all nodes would be infected given enough time (${I}_{\infty }=N$ as $t\to \infty )$. With interventions implemented, the HIV transmission could reach the endemic equilibrium and stop at a certain time point $T$, meaning ${I}_{T}={I}_{T+1}={I}_{T+2}=\ldots ={I}_{\infty } < N$.

### Network Generation Algorithms

BA Scale-Free Network model and ER Random Graph model were used to generate social networks. BA Scale Free model has a preferential attachment mechanism^57^. The network begins with a connected network of $${m}_{0}$$ ($${m}_{0}=20)$$ nodes. New nodes are being added iteratively. Each new node is connected to $$m\,(m\le {m}_{0},m=5\,)$$ existing nodes. The probability of an existing node to be connected is proportional to its degree. In ER Random Graph model^58^, the numbers of nodes and edges are set as $$N$$ and $$M$$, the probability ($$p=0.0004$$) of the existence of an edge between two nodes is the same for all node pairs. For both BA and ER models, if a new edge form odd cycles in the network, we discard it. Therefore, the generated sexual relationship network is a two-mode network^59,60^, which is reasonable for this study as we only consider heterogeneous sexual relationships.

### Target Node Identification Methods

The degree centrality of a node denotes the number of edges connected to this node in a single-layer network. In the proposed 2-layer network framework, the degree $D({v}_{i})$ is the degree of node ${v}_{i}$ in corresponding integrated network ${G}_{integrated}$. Betweenness centrality measures the influence of a node on the transfer of items (e.g. information, virus, products, etc.) in the network^37^. The betweenness centrality $B({v}_{i})$ in the 2-layer network is the proportion of shortest paths (between two nodes other than ${v}_{i}$) traversing through the node ${v}_{i}$ in the integrated network ${G}_{integrated}$:

$$B({v}_{i})=\sum _{{v}_{i}\ne {v}_{u}\ne {v}_{t}\in V}\frac{{\delta }_{{v}_{u},{v}_{t}}({v}_{i})}{{\delta }_{{v}_{u},{v}_{t}}},$$

where ${\delta }_{{v}_{u},{v}_{t}}$ is the number of the shortest path between ${v}_{u}$ and ${v}_{t}$. ${\delta }_{{v}_{u},{v}_{t}}({v}_{i})$ is the number of the shortest path between ${v}_{u}$ and ${v}_{t}$ through ${v}_{i}$.

## Electronic supplementary material

Supplementary materials
