# Supplementary material for: Modeling the Intervention of HIV Transmission across Intertwined Key Populations
Source: Sci Rep. 2018 Feb 5;8:2432. doi: 10.1038/s41598-018-20864-6 (PMC5799486; doi:10.1038/s41598-018-20864-6)
Supplement: Supplementary file 1 — Supplementary materials [file 41598_2018_20864_MOESM1_ESM.pdf]

Supplementary materials

Modeling the Intervention of HIV Transmission across Intertwined Key Populations

Lu Zhong[1], Qingpeng Zhang[1,2*,] Xiaoming Li[3]

1. Department of Systems Engineering and Engineering Management, City University of Hong Kong, Kowloon, Hong Kong SAR, China.
2. Shenzhen Research Institute of City University of Hong Kong, Shenzhen, Guangdong, China.
3. Arnold School of Public Health, University of South Carolina, Columbia, SC, USA.

* qingpeng.zhang@cityu.edu.hk

(This supplementary document presents the additional experiments to support the conclusion of the main manuscript. Here, we target at bridge nodes first, and then non-bridge nodes during the interventions. These experiments were presented in the original main manuscript. We moved it to supplementary materials to control the length and focus on the main manuscript. We also did further experiment with a varying transmission rate.)

From the results presented in the manuscript, we found that it is critical to reaching the segregation point to limit HIV transmissions within one community. Therefore, we conducted another set of simulations with the latter four intervention strategies (IR_bridge, ID_bridge, IB_bridge, and IBc_bridge), see in Table S.1, which isolated all bridge nodes first, and then the rest of others. The simulation results reinforced our finding of bridge nodes' critical role in HIV transmissions across communities. As shown in Figure S.1, all strategies could achieve the segregation point with smaller values of α than those in previous experiments. In these simulations, IBc did not show superior performance because we had already focused on isolating bridge nodes for all intervention strategies, the roles of other topological properties became more important than cross-layer paths. It is worth noting that the actual percentage of bridge nodes was 0.01. But we could actually achieve

the segregation point with a degree of isolation smaller than 0.01 (as shown in Figure S.1 and Figure 3b). This finding indicated that we do not have to isolate all bridge nodes in order to segregate the HIV transmissions. This is because we only need to isolate one bridge node for each bridge edge to cut the corresponding edge. This also explained the observation that IR_bridge reached the segregation point with a smaller degree of isolation as compared with other metric-based strategies.

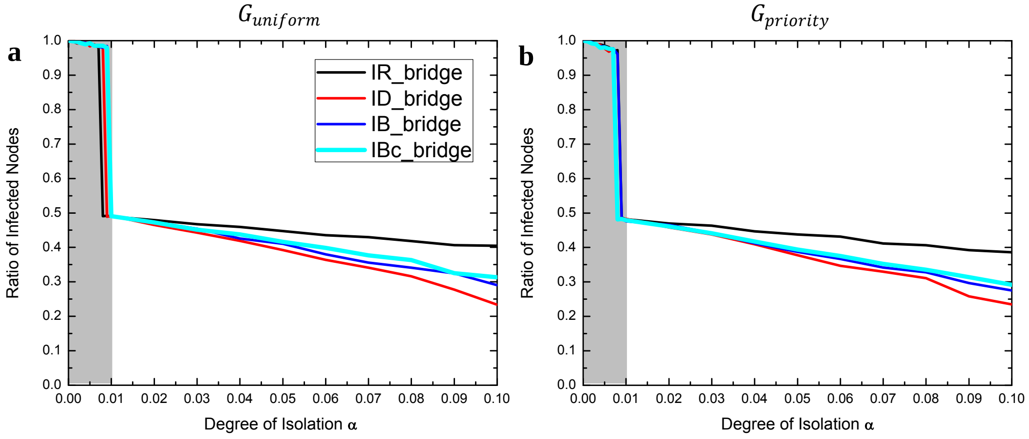

Figure S.1. The ratios of infected nodes of intervention strategies (IR_bridge, ID_bridge, IB_bridge, IBc_bridge) in endemic equilibrium with varying degrees of isolation $\boldsymbol{\alpha}$ ($\mathbf{0 \leq \alpha \leq 0.1}$). The dashed area has a higher resolution of α (0.001) because segregations occurred here. (a) $G_{uniform}$. (b) $G_{priority}$.

Table S.1. Intervention strategies isolating bridge nodes first.

Strategy	Descriptions
IR_bridge	Isolate bridge nodes first and then non-bridge nodes randomly.
ID_bridge	Isolate bridge nodes based on degree centrality first, and then non-bridge nodes based on degree centrality (descending order).
IB_bridge	Isolate bridge nodes based on betweenness centrality first, and then non-bridge nodes based on betweenness centrality (descending order).
IBc_bridge	Isolate bridge nodes based on cross-layer betweenness centrality first, and then non-bridge nodes based on cross-layer betweenness centrality (descending order).

To account for the randomness of human behaviors, we also did another set of experiments to allow the transmission rate β_1 and β_2 to vary for different edges following a Gaussian distribution: $\beta_1 \sim N(0.1, \sigma^2)$, $\beta_2 \sim N(0.05, \sigma^2)$. Simulations with varying transmission rates led to similar results as what we reported in the main manuscript, shown in Figure R.2.

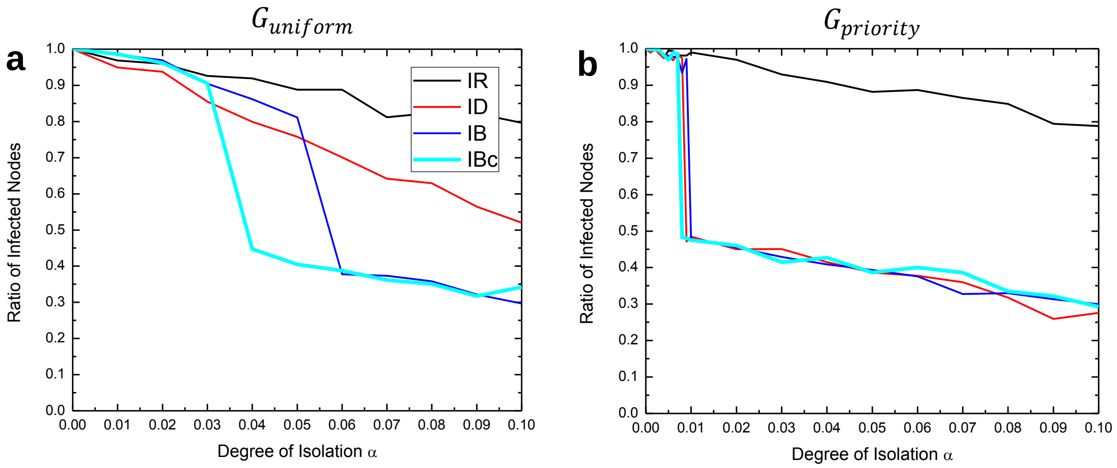

Figure S.2. The ratios of infected nodes of intervention strategies (IR, ID, IB, IBc) at the endemic equilibrium with varying degrees of isolation $\boldsymbol{\alpha}$ $(\mathbf{0 \leq \alpha \leq 0.1})$ under varying transmission rates. $\beta_1 \sim N(0.1,0.01)$, $\beta_2 \sim N(0.05,0.01)$. (a) $G_{uniform}$. (b) $G_{priority}$.
